# Revisiting the dengue epidemic of 2011 in Paraguay: molecular epidemiology of dengue virus in the Asuncion metropolitan area

**DOI:** 10.1186/s12879-021-06487-9

**Published:** 2021-08-07

**Authors:** Alejandra Rojas, Adriana Moreira Soares, Laura Patricia Mendoza, María Eugenia Acosta, Laura Aria, Malvina Páez, Lilian Herebia, María Asunción Vallejos, Yvalena de Guillén, Victor Hugo Aquino

**Affiliations:** 1 Department of Production, Health Sciences Research Institute, National University of Asuncion, San Lorenzo, Paraguay; 2 Laboratory of Virology, Department of Clinical Analyses, Toxicology and Food Sciences, School of Pharmaceutical Sciences of Ribeirao Preto, University of Sao Paulo, Ribeirão Preto, Brazil; 3 Department of Public Health, Health Sciences Research Institute, National University of Asuncion, San Lorenzo, Paraguay; 4 Emergency Department, Central Hospital of the Institute of Social Welfare, Asunción, Paraguay

**Keywords:** Dengue virus, Molecular epidemiology, Envelope glycoprotein, Genetic diversity, Phylogenetic relationship

## Abstract

**Background:**

Dengue is one of the most important re-emerging viral diseases and the most common human arthropod-borne viral infection worldwide. Any of the four *Dengue virus* serotypes (DENV-1 to 4) can cause asymptomatic infections or clinical manifestations that range in severity from a mild, self-limited illness, to a severe disease characterized by a shock syndrome that can lead to death. Paraguay suffers periodic epidemic outbreaks of dengue since 1988 when the DENV-1 was introduced in the country. Epidemics caused by all four serotypes have been reported and the country. Although dengue is endemic in Paraguay, few studies have described the molecular epidemiology of DENV in the country, which is important to understand the local and global spread, as well as the evolution of this pathogen.

**Methods:**

This was a cross-sectional study of a convenience sample. Suspected dengue patients of any age were recruited from the Emergency Laboratory of the Central Hospital of the Institute of Social Welfare, Asuncion, Paraguay, from February to June of 2011. A DENV antigen test was used to confirm the infection. The protein E gene sequences of isolated viruses were sequenced for phylogenetic analysis.

**Results:**

Dengue was confirmed in 55.1% of the participants (n = 98/178). The most frequent clinical findings were fever, headache, and myalgia. Identity analyses of the protein E gene sequence of 56 viruses isolated showed the circulation of DENV-1 (n = 45) and DENV-2 (n = 11) in the Asuncion metropolitan area in 2011. Molecular epidemiology analyses suggest that DENV-1 was introduced into Paraguay from Argentina, while the DENV-2 from Brazil, replacing previous virus lineages.

**Conclusions:**

We have analyzed the molecular epidemiology of DENV-1 and DENV-2 isolated in Paraguay in 2011. We found strong evidence that DENV-1 was introduced into Paraguay from Argentina, while the DENV-2 from Brazil, replacing previous virus lineages. Molecular epidemiology studies are of great interest to analyze the dynamic of DENV spread, which are useful for early implementation of containment measures to reduce the risk of explosive epidemics caused by this virus.

**Supplementary Information:**

The online version contains supplementary material available at 10.1186/s12879-021-06487-9.

## Background

Dengue is the most common human arthropod-borne viral infection, affecting at least 128 countries in tropical and subtropical regions around the world, with an estimate of 3.97 billion people at risk of infection with the *Dengue virus* (DENV). The incidence of dengue has grown dramatically worldwide in recent decades, with an estimate of 390 million dengue infections per year, of which 96 million manifests clinically with any severity of disease [1]. Dengue viruses are traditionally grouped into four different serotypes (DENV-1 to 4) [2–4], which can cause asymptomatic infections or clinical manifestations that range in severity from a mild, self-limited illness, to a severe disease characterized by a shock syndrome that can lead to death [5]. DENV belongs to the *Flaviviridae* family, *Flavivirus* genus. DENV has a positive-sense, single-stranded RNA genome that is ∼ 11 kb in length and encodes a single polyprotein that is cleaved by viral and cellular proteases in the structural capsid (C), pre-membrane (prM), and envelope (E) proteins, in addition to seven non-structural proteins (NS1, NS2A, NS2B, NS3, NS4A, NS4B, and NS5).

In Paraguay, the first dengue cases were detected in 1988–1989 when DENV-1 caused an epidemic outbreak with more than 40,000 reported cases, affecting mainly the city of Asuncion, the capital of the country [6, 7]. The DENV-1 introduced in the country was closely related to viruses circulating in Brazil, Colombia, Mexico, and the Caribbean region [8]. Ten years later, between 1999 and 2000, the second outbreak of DENV-1 occurred, when the virus spread throughout Asuncion and the Central, Alto Parana, and Amambay Departments, with an estimated 100,000 cases [7, 9]. DENV-2 was introduced in the country in 2001 and co-circulated with DENV-1; only 38 cases were reported in that year [10]. In 2005, a new lineage of DENV-2 was introduced in Paraguay [11]. DENV-3 was detected for the first time in 2002 when this serotype co-circulated with DENV-1 and DENV-2; 1800 cases were reported in that year [12]. Phylogenetic analyses suggest that the DENV-3 was introduced into Paraguay from different regions of Brazil [13–16]. The circulation of DENV-3 continued in the following years and, in 2007, it was responsible for a great epidemic, affecting most of the country’s territory, with 28,182 reported cases and, for the first time, deaths (n = 17) due to dengue was observed in Paraguay [17]. Although DENV-3 was responsible for the 2007 outbreak, DENV-2 was also circulating at that moment [18]. After an inter-epidemic period, more than 20,000 cases and 15 deaths were reported from October of 2009 to December of 2010 [19]. The DENV-1, DENV-2, and DENV-3 circulated during that epidemic outbreak. In 2011, during the co-circulation of DENV-1 and DENV-2, Paraguay suffered the most severe dengue epidemic observed until then, with 42,264 cases and 62 deaths [19].

Although Paraguay is a dengue-endemic country, few studies described the molecular epidemiology of DENV in the country. Epidemiological studies are important to understand the local and global spread, as well as the evolution of this pathogen. In this study, we analyzed the molecular epidemiology of DENV-1 and DENV-2 isolated in 2011 in the Asuncion metropolitan area to improve the information in the molecular epidemiology of these viruses in the South cone of South America.

## Methods

### Study place, patient recruitment and clinical samples

This was a cross-sectional study of a convenience sample comprising 178 participants recruited from February to June of 2011. Suspected dengue patients of any age were recruited from the Emergency Laboratory of the Central Hospital of the Institute of Social Welfare (CH-ISW), Asuncion, Paraguay. The CH-ISW is a tertiary care hospital that provides medical attention to patients from Asunción and the metropolitan area, which includes several neighboring cities. All patients had a dengue NS1 antigen test requested by the attending physicians. Trained staff of the CH-ISW were responsible for the dengue NS1 tests (Standard Diagnostics, Inc., Korea), which was part of the routine laboratory tests performed during the epidemic period. An aliquot (~ 2 mL) of serum sample of all suspected dengue cases were immediately transported to the Department of Production of the Health Sciences Research Institute (HSRI), National University of Asuncion (﻿UNA), in refrigerated boxes and then stored at − 70 °C until use. Demographic and clinical data, including the result of the dengue NS1 antigen test, were obtained retrospectively from the patient’s medical records. The informed consent was not requested to the patients because this is a descriptive and retrospective study in which privacy and confidentiality of clinical data and the subjects involved were assured. The residence address of patients was mapped using Google Earth 6.2.2.6613 (Google Inc., USA, http://earth.google.com). Asuncion, the capital of Paraguay, is on the western region of Paraguay (25°16ʹ S, 57°40ʹ W), at the left side of the Paraguay River, on the border with Argentina. Asuncion metropolitan region includes an urban area of 1014 km^2^ with an estimated population in 2011 of 2,597,588 inhabitants [20]. This region has a humid subtropical climate with an average annual temperature of 23 °C and average annual precipitation of 1700 mm. The Ethics Committee of the HSRI-UNA approved this study (Permit Nº P15/2011). The inclusion criterion was patients with DENV infection confirmed by the NS1 test. The exclusion criterion was patients with negative NS1 tests.

### Virus isolation

A monolayer of C6/36 cells, contained in a 25 cm^2^ flask in L15 medium, was inoculated with 10 µL of serum samples. Cells were incubated at 28 °C for at least 7 days (passage 0). An aliquot of 50 µL of the inoculated cell culture supernatant was used to inoculate a second flask containing C6/36 cells (passage 1), which were incubated for further 7 days. Virus isolation was confirmed by detection of the viral genome in the supernatant of the cell culture (passage 1) by real-time reverse transcription followed by a polymerase chain reaction (RT-PCR) as described previously [21].

### Real-time RT-PCR for detection of DENV genome

A real-time RT-PCR method was used to confirm the DENV infection of the patients and the cell cultures as previously described [21].

### Protein E gene amplification and sequencing

#### Viral RNA purification

The viral RNA was purified from 200 µL of cell culture supernatant using the AxyPrep Body Fluid Viral DNA/RNA Miniprep Kit (Axygen Biosciences, EUA), following manufacturer’s recommendations. The RNA was eluted with 60 µL of elution buffer and stored at − 80 °C until use.

#### DENV protein E gene amplification

The protein E gene of DENV was amplified by a conventional RT-PCR method. The reaction mixture for the reverse transcription of the viral RNA contained 23 μL of RNA, 200 ng of random primers (Invitrogen, USA), 0.25 mM dNTPs mix (Invitrogen, USA), 80U of RNAse inhibitor (RNAseOUT, Invitrogen, USA), 400U of M-MLV Reverse Transcriptase (USB, USA) and 8 μL 5× buffer (250 mM Tris-HCl [pH 8.3], 37 mM KCl, 15 mM MgCl_2_) in a final volume of 40 μL. The mixture was incubated at 25 °C for 10 min, followed by incubation at 37 °C for 4 h, and a final incubation of 5 min at 85 °C. The PCR reaction mixture contained 1 μL of the cDNA, 0.2 mM dNTP, 0.3 mM of each primer for DENV-1, DENV-2 and DENV-3 (Table 1), 1.5U of PlatinumTaq DNA Polymerase High Fidelity (Invitrogen, USA), 5 μL of 10× buffer (600 mM Tris-SO_4_ [pH 8.9], 180 mM ammonium sulfate), and 2 mM of MgSO_4_ in a final volume of 50 μL. The amplification was performed using the MyCycler™ Thermal Cycler (BIO-RAD, USA). The reaction mixture was heated at 94 °C for 2 min followed by 45 amplification cycles of − 94 °C for 10 s, 56 °C for 1 min, 68 °C for 1 min, and a final extension at 68 °C for 7 min. Amplification products were detected by 1.8% gel electrophoresis stained with GelRed (Biotium Inc, USA). The amplification products were purified from the agarose gel using the QIAquick Gel Extraction Kit (Qiagen, USA), following the manufacturer’s instructions.

**Table 1:** Primers used in the RT-PCR assay for amplification of the E protein gene of DENV

Virus	Primers	Sequence (5ʹ- 3ʹ)	Position	GenBank Nº	References
DENV-1	d1s3	AAACGTTCCGTSGCACTGGC*	704–709	MT447147	[22]
	d1a17	CCAATGGCYGCTGAYAGTCT*	2540–2559		
DENV-2	ES(D2)B	GGCATACACCATAGGAACGAC	858–878	MN018365	Designed in this study
	EC(D2)B	AGGGGATTCTGGTTGGAACTT	2518–2538		
DENV-3	ES(D3)	GCCCTATTTCTTGCCCATTACA	845–866	MH823209	[13]
	EC(D3)	CCGCACACTCCATTCTCCCAA	2560–2580		

^*^S = G + C; Y = C + T

#### Nucleotide sequencing and similarity analysis of the E protein gene sequences

The PCR DNA fragments were sequenced using the ABI PRISM BigDye Terminator v3.1 Cycle Sequencing kit (Applied Biosystems, EUA), following the manufacturer’s recommendations. The sequencing reaction was subjected to capillary electrophoresis in the ABI PRISM 3500 DNA Sequencer (Applied Biosystems, EUA). Both strains of each DNA fragment were sequenced at least three times using PCR and walking primers. Protein E gene sequences generated in this study were deposited in the GenBank, under the Accession numbers: KF419412-32. Similarity analyses of the sequences were performed with the CLC Main Workbench software (QIAGEN, USA) and the Basic Local Alignment Search Tool (BLAST, National Center for Biotechnology Information, https://www.ncbi.nlm.nih.gov/, USA).

### Phylogenetic analysis

The E gene sequences of the DENV-1 (n = 15) and DENV-2 (n = 6), obtained in this study, were aligned with the E gene sequences of representative DENV-1 (n = 381) and DENV-2 (n = 702) isolated worldwide. The nucleotide sequences of the viruses were retrieved from the GenBank until June of 2019. The alignment was performed with the CLC Main Workbench software (QIAGEN, USA). The Galaxy, an open-source, web-based platform, was used for the phylogenetic analysis [23]. The IQ-TREE program was used for phylogenetic reconstruction [24]. The selection of the optimal model of sequence evolution was performed with the ModelFinder program [24]. To compute the support of phylogenetic groups in the Maximum-Likelihood based trees, the ultrafast bootstrap (UFBoot) approximation approach was used [25].

## Results

Suspected dengue patients (n = 178), managed at the emergency unit of the Central Hospital of the Institute of Social Welfare (CH-ISW) in Asuncion, Paraguay, were recruited in this study. A DENV NS1 antigen test confirms dengue diagnosis in 98/178 (55.1%) patients. Demographic characteristics and symptoms of dengue patients are shown in Table 2. The most frequent clinical findings were fever, headache, and myalgia. We did not find an association between age or gender with the presence of dengue in these patients.

**Table 2:** Demographic characteristics and symptoms of dengue patients (n = 98)

Characteristic	Frequency	%
Gender		
Male	49	50.0
Female	49	50.0
Age		
2–15	29	29.6
16–31	28	28.6
32–47	19	19.4
48–63	10	10.2
64–78	2	2.0
No data	10	10.2
Symptoms		
Fever	92	93.9
Headache	68	69.4
Myalgia	67	68.4
Arthralgia	52	53.1
Retro-orbital pain	50	51.0
Nausea	38	38.8
Abdominal pain	22	22.4
Vomit	15	15.3
Petechiae	9	9.2
Exanthema	6	6.1

A real-time RT-PCR) test detected the presence of DENV genome in the serum sample of 93/98 (94.9%) dengue patients. An aliquot of those samples was inoculated in C6/36 cells for tentative virus isolation. Analysis of the cell culture supernatants by real-time RT-PCR confirmed the virus isolation from 78/93 (83.9%) serum samples. To identify the virus serotypes, the protein E gene of the isolated viruses was amplified by conventional RT-PCR, using primers for the DENV-1, DENV-2, and DENV-3 (Table 1). Primers for the DENV-4 were not used because this virus was still not introduced in the country by that time. We were able to obtain a sufficient amount of PCR products from 56 isolates for nucleotide sequencing. Similarity analyses of the protein E gene sequences, using the Basic Local Alignment Search Tool (BLAST), showed that isolated viruses belong to DENV-1 (n = 45) and DENV-2 (n = 11).

**Fig. 1:**
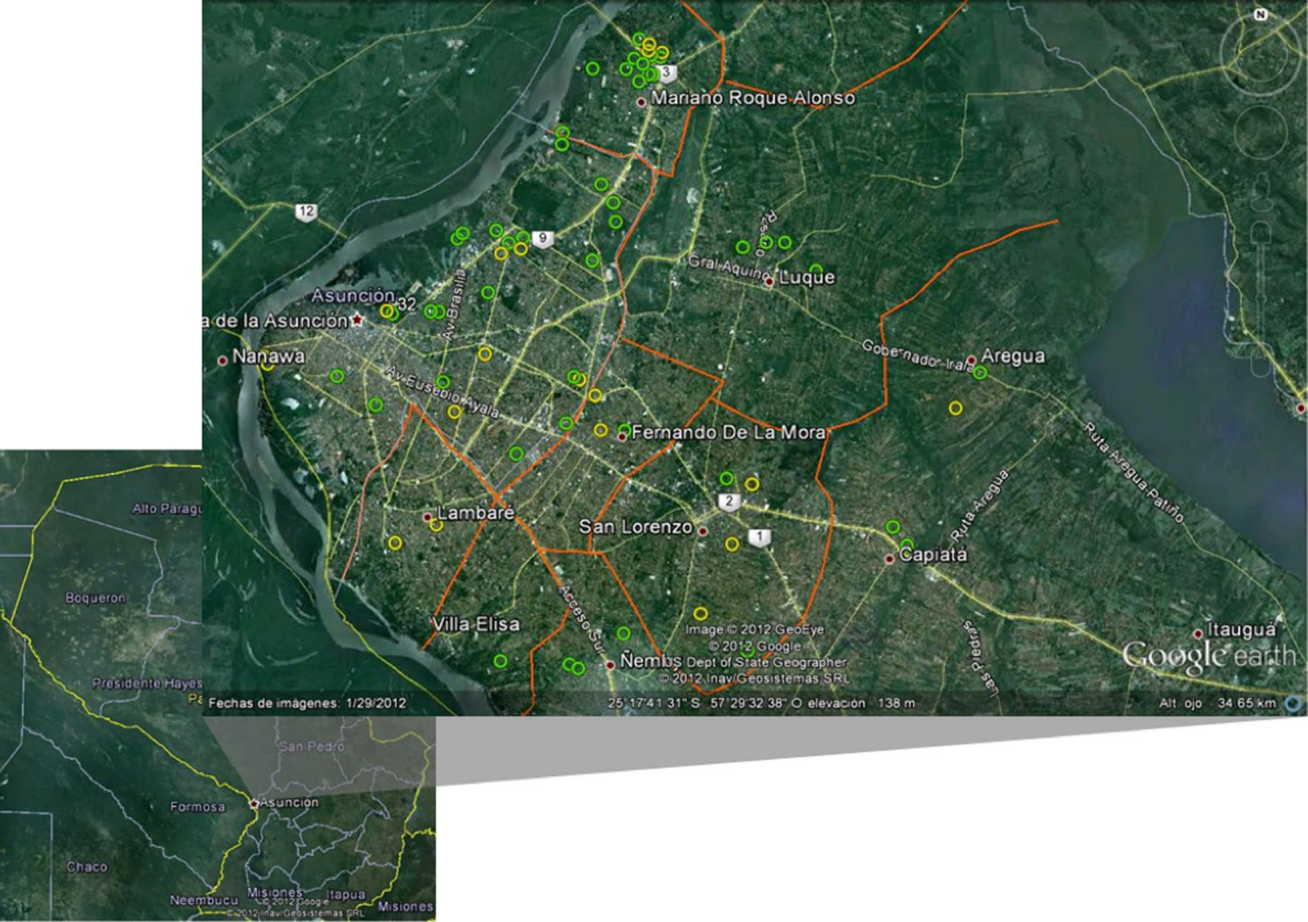
Geographic distribution of dengue cases in the Asuncion metropolitan area. The open green and open yellow circles represent the DENV-1 and DENV-2 cases, respectively. The dengue cases outside the Asunción metropolitan area are not shown in the map (Villa Hayes in the West; Emboscada in the Northeast; and Itá in the Southeast). The residence address of patients was mapped using Google Earth 6.2.2.6613 (Google Inc., USA). The map was based on a satellite picture available in Google Earth (http://earth.google.com)

The geographic distribution of the dengue cases detected in this study is shown in Fig. 1. The dengue cases were distributed mainly in Asuncion and the metropolitan area, which includes the cities of Lambaré, Mariano Roque Alonso, Villa Elisa, Fernando de la Mora, San Lorenzo, Capiatá, Ñemby, and Areguá. Although a higher number of DENV-1 cases were detected, both the DENV-1 and DENV-2 cases were evenly distributed in the Asuncion metropolitan area. Few patients belonged to cities outside the Asuncion metropolitan area, from Villa Hayes at the West (n = 1), Emboscada at the Northeast (n = 1), and Itá (n = 1) at the southeast of Asuncion.

### Phylogenetic analysis

To investigate the genetic relationships among the DENV-1 and DENV-2 isolates from Paraguay and other countries in South America, we constructed phylogenetic trees using the Maximum-Likelihood method.

**Fig. 2:**
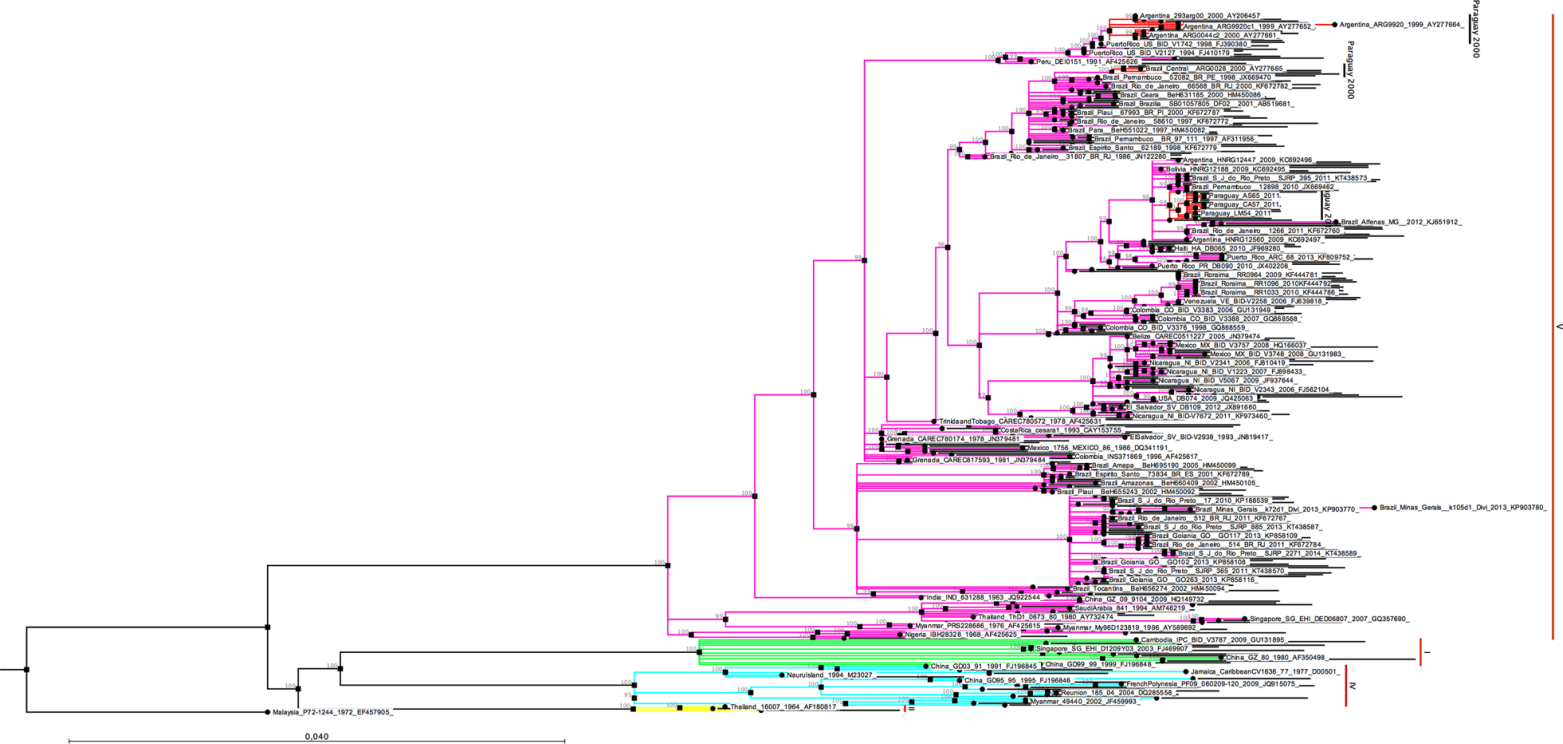
DENV-1 phylogenetic tree. Phylogenetic relationships among the E protein gene sequences of DENV-1 were resolved using a Maximum-Likelihood tree inferred with IQ-TREE under the GTR + R4 model of nucleotide substitution. Robustness of tree topology was assessed with 1000 bootstrap replicates. Nodes with bootstrap values ≥ 95% are shown. A Sylvatic isolate (Malaysia_P72-1244_1972_EF457905) of the genotype III was used to root the tree. The tree was visualized with the CLC Sequence Viewer 8 program (QIAGEN, USA)

**Fig. 3:**
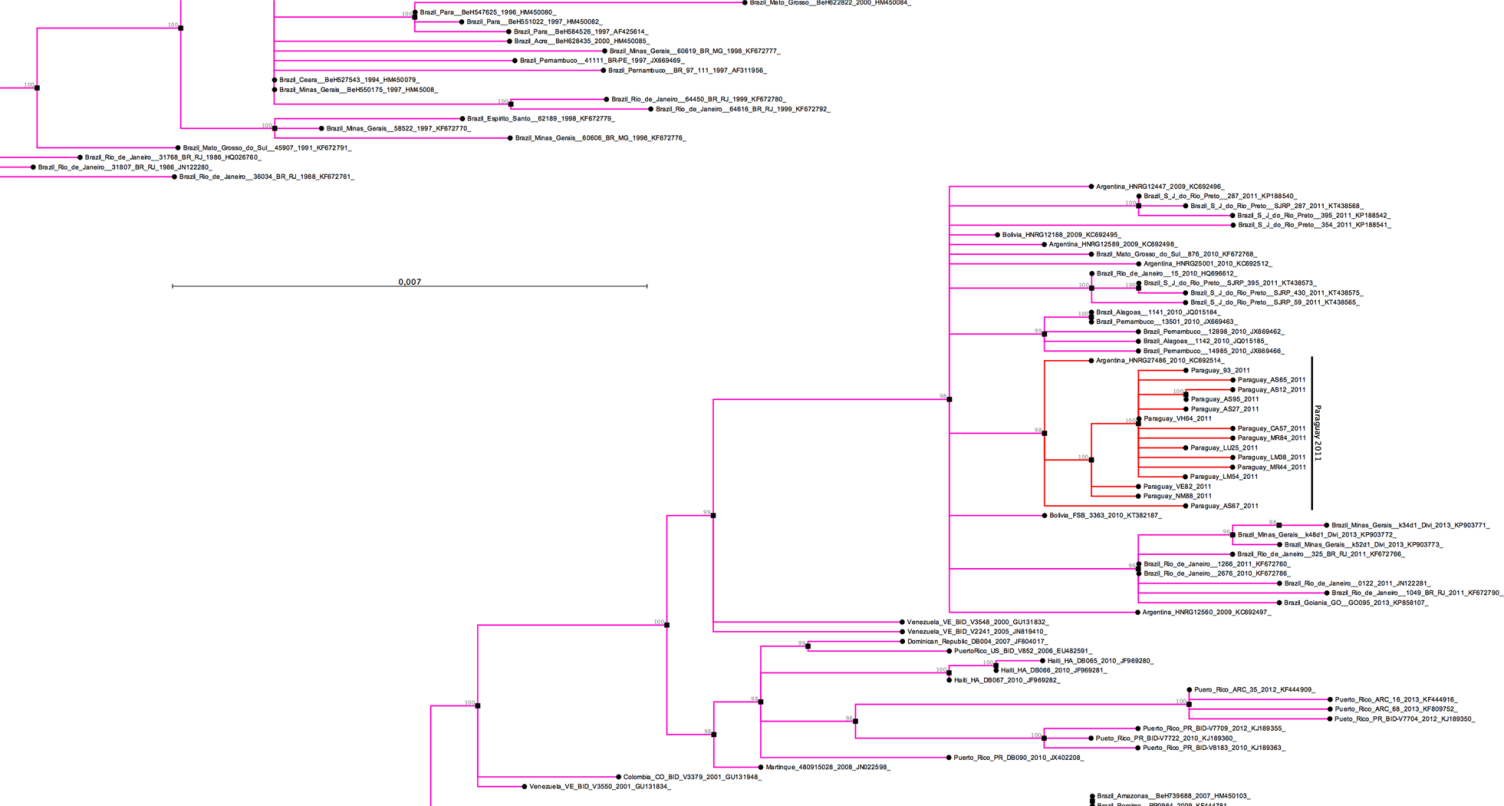
The Paraguayan cluster of DENV isolated in 2011. Zoom of the clade where the DENV-1 isolated in the Asuncion metropolitan area in 2011 are grouped

Comparison of the protein E gene sequences of the 45 DENV-1 isolated in the Asuncion metropolitan area showed an identity among them ranging from 99.53 to 100%; 31 isolates showed identical sequences. For phylogenetic analysis, the non-identical nucleotide sequences of 15 DENV-1, isolated during this study, were aligned with the protein E gene sequence of other DENV-1 (n = 381) isolated worldwide (Additional file 1). The phylogenetic tree, constructed based on the nucleotide alignment, shows the clustering of DENV-1 strains into four, highly supported, monophyletic genotypes (I, II, IV, and V) circulating among humans (Fig. 2). Members of the Sylvatic DENV-1 genotype III were represented by a single sequence (Malaysia_P72-1244_1972_EF457905), which was used to root the tree. The DENV-1 isolated in Paraguay grouped within the genotype V, which includes viruses isolated in the Americas, the Caribbean region, West Africa, and Asia. The DENV-1 isolated in Paraguay in 2011 grouped in a different cluster from those isolated in 2000, suggesting the introduction of a new virus lineage in the country. The Paraguayan DENV-1 of 2011 grouped with viruses isolated in Argentina, Bolivia, Brazil, and Venezuela. This cluster was probably originated from viruses circulated in Venezuela between 2000 and 2005. The virus then spread to Argentina, Bolivia, Paraguay, and Brazil. The Paraguayan isolates were closely related to a virus isolated in Argentina in 2010 (GenBank Nº HNRG27486), suggesting that the DENV-1 was introduced into Paraguay from Argentina (Fig. 3).

**Fig. 4:**
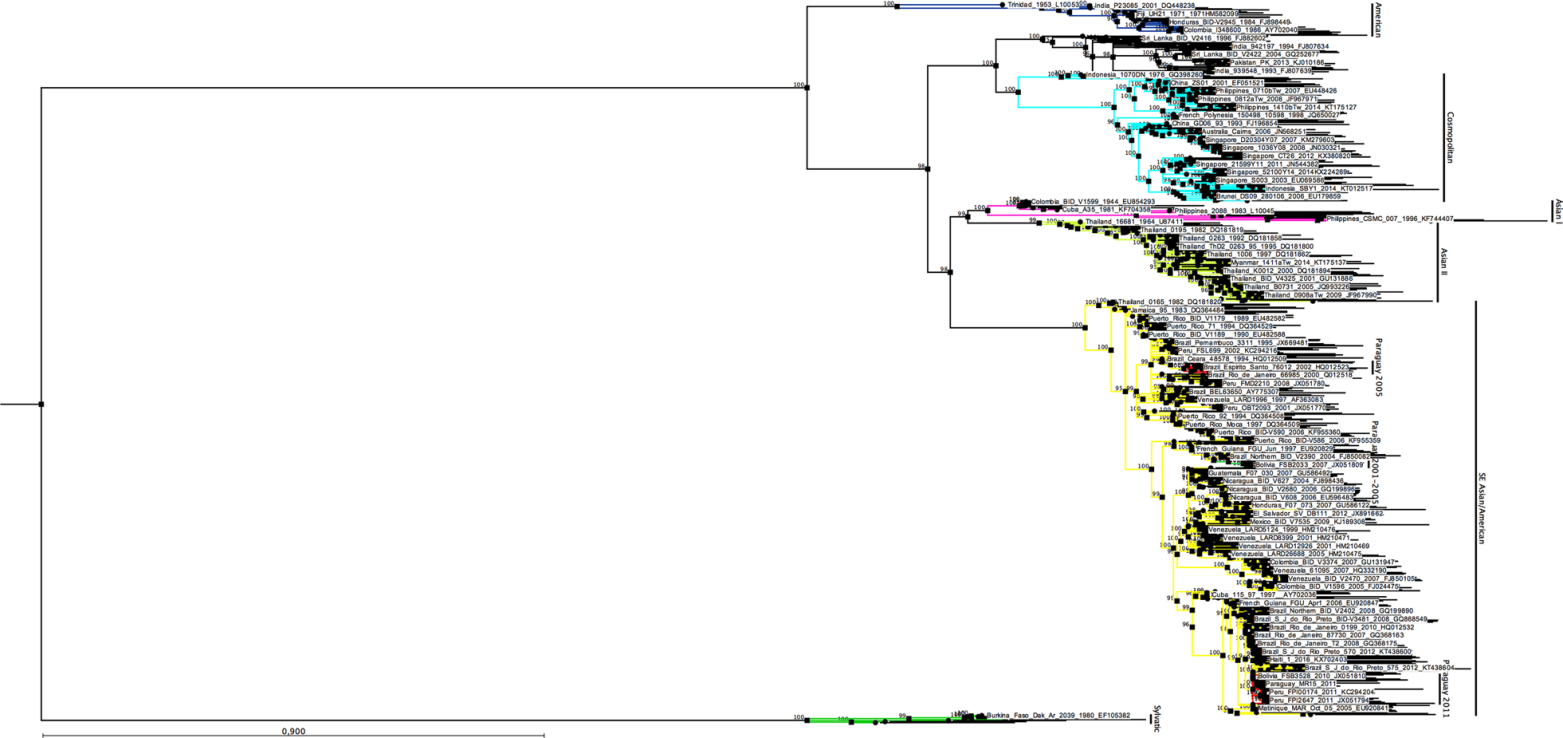
DENV-2 phylogenetic tree. Phylogenetic relationships among the E protein gene sequences of DENV-2 were resolved using a Maximum Likelihood (ML) tree inferred with IQ-TREE under the GTR + R4 model of nucleotide substitution. Robustness of tree topology was assessed with 1000 bootstrap replicates. Nodes with bootstrap values ≥ 95% are shown. The Sylvatic isolates were used to root the tree. The tree was visualized with the CLC Sequence Viewer 8 program (QIAGEN, USA)

**Fig. 5:**
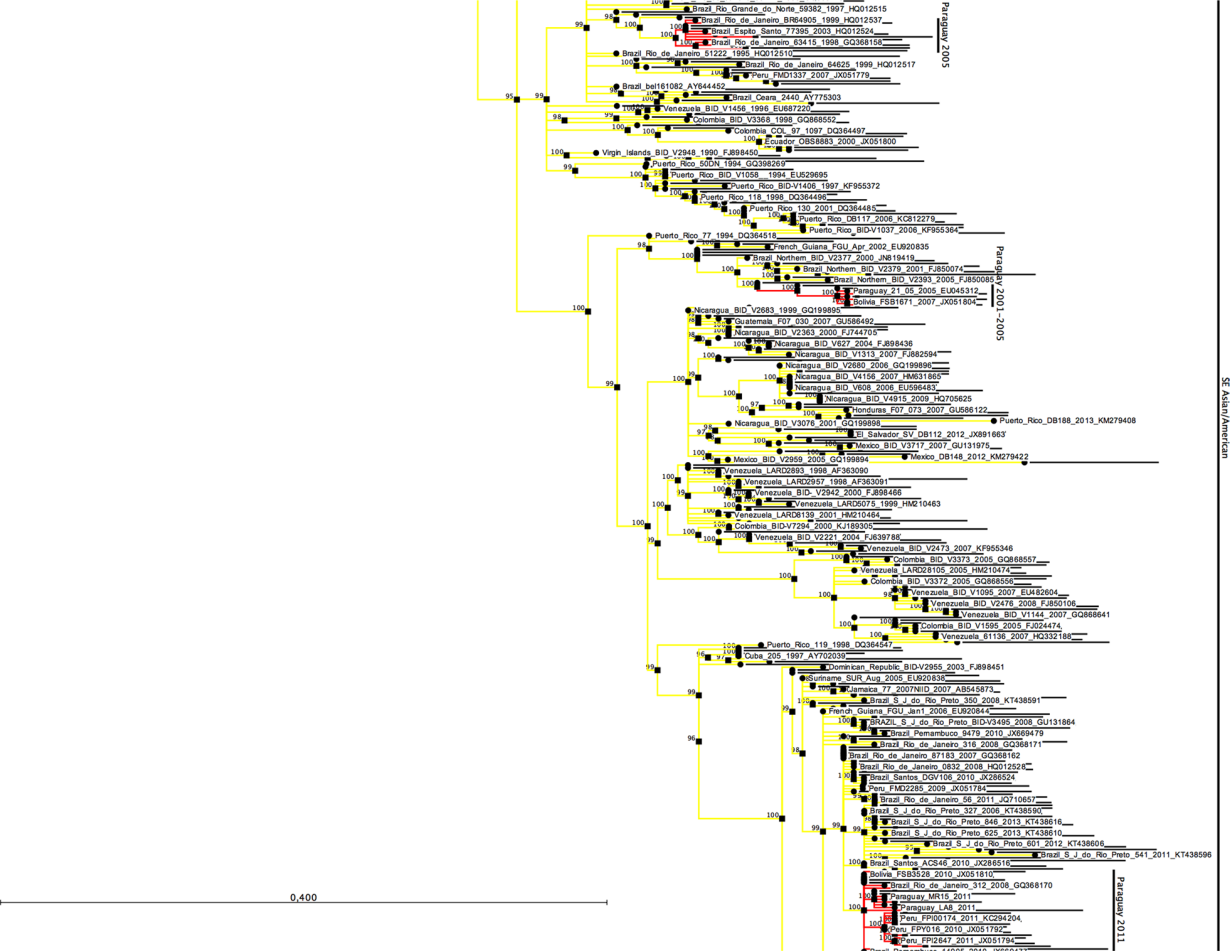
The Paraguayan clusters of DENV-2. Zoom of the clusters where the DENV-2 isolated in 2001, 2005, and 2011 are grouped

The E protein gene sequences of DENV-2 isolated from 11 patients in the metropolitan area of Asuncion showed an identity among them ranging from 99.73 to 100%. Six non-identical sequences of DENV-2, isolated in this study, were aligned with the E protein gene sequence of DENV-2 (n = 702) isolated worldwide (Additional file 2). The phylogenetic tree, constructed based on the nucleotide alignment, shows the clustering of DENV-2 strains into the six characteristic groups or genotypes: American, SE Asia/American, Cosmopolitan, Asian I, Asian II, and Sylvatic (Fig. 4). All the DENV-2 isolated in Paraguay grouped within the SE Asia/American genotype. The DENV-2 isolated in Paraguay in 2011 grouped in a different cluster from those isolated in 2001–2005, suggesting the introduction of a new virus lineage in the country. The Paraguayan DENV-2 isolated in 2011 were located in a clade including viruses from Peru and Bolivia isolated between 2009 and 2011, and the Southeast region of Brazil, the oldest one isolated in 2007–2008 in Rio de Janeiro, which was probably the place where this clade was originated (Fig. 5).

## Discussion

In this study, we have identified the circulation of DENV-1 and DENV-2 in the Asuncion metropolitan area during the epidemic of 2011, which agrees with data reported by the national health authorities [26]. However, a greater prevalence of DENV-1 was observed in this study, while the national health authorities found a greater prevalence of DENV-2. In 2011, the dengue epidemic affected almost the whole country, with 42,264 cases reported. Our study was restricted to the circulation of DENV mainly in the Asuncion metropolitan area, which may explain the difference in serotype prevalence found in this study when compared with those reported by the national health authorities. This epidemic appeared after 11 years of the last one caused by DENV-1, when approximately 27,000 to 100,000 dengue cases may have occurred in the Asuncion metropolitan area [7, 9]. In addition, a limitation of our study was the use of convenience sampling, which may have influenced the prevalence of DENV serotypes found in this study. Asuncion and the cities of the metropolitan area have experienced a demographic growth in the last years. In 2000, the Central Department, where the cities of the Asunción metropolitan area belong, had a population of 1,342,589 inhabitants, while in 2011, this number has reached to 1,811,838 inhabitants [27]. Besides, when DENV-2 was introduced in the country in 2001, and then in 2005, a few numbers of dengue cases were reported [11]. These data explain the high number of susceptible individuals, which might have favored the dengue epidemic in 2011 when co-circulation of both DENV-1 and DENV-2 were detected.

We have used the Maximum-Likelihood method to analyze the phylogenetic relationship of DENV isolated in this study. The Maximum-Likelihood consensus tree showed the expected topology and classical segregation of the sequences into five genotypes for DENV-1 [28, 29] and six genotypes DENV-2 [29, 30], respectively. The Paraguayan DENV-1 isolates were all grouped with viruses of the genotype V, which also includes most of the isolates from South America, Central America, and the Caribbean region, as well as isolates from India, East Africa, West Africa, and Southeast Asia. Our phylogenetic analysis shows that the DENV-1 of the 2000 and 2011 epidemics grouped in different clusters, indicating the introduction of a new virus lineage in the country in 2011. The cluster where the DENV-1 isolated in the Asuncion metropolitan area are grouped, arose probably in Venezuela, around the late 1980s, and represents the most successfully disseminated viral lineage in the American continent [31]. This lineage of DENV-1 spread from Venezuela to several countries, including Argentina, and from there to the Brazilian Northeastern region [31]. Our phylogenetic analysis showed a close relationship of the DENV-1 isolated Paraguay in 2011 with a DENV-1 isolated in Argentina in 2010, strongly suggesting that this lineage was disseminated also from Argentina to Paraguay. This finding is further supported by epidemiological data. Between August to December of 2008, the Paraguayan health authorities have not detected any suspected dengue cases, suggesting the virus was not circulating in the country during that period of time [32]. In the first months of 2009, new dengue cases were reported in Paraguay, suggesting a recent introduction of the virus in the country [33]. In the meantime, between October 2008 and June 2009, an outbreak of DENV-1 was first detected in Bolivia, spreading then to Argentina. The DENV-1 was initially detected in the Northern provinces of Argentina and, finally, reaching the capital, Buenos Aires and the metropolitan area, where the highest number of cases was reported in that country [34, 35]. There is a long history of Paraguayans emigrating to Argentina seeking better economic opportunities, which in addition to the commercial and tourism trips, lead to an intense movement of individuals between both countries that might have been responsible for the introduction of the DENV-1 from Argentina to Paraguay in 2009. The introduction of DENV-1 from Bolivia is least likely because very few travels between Paraguay and Bolivia occur due to economical and geographical issues. The spread of DENV between Paraguay and Argentina has been described previously; however, in the opposite direction. Previous studies have shown that the DENV-1 and DENV-3 have been disseminated from Paraguay to Argentina in 2000 and 2007, respectively [36, 37]. A spatiotemporal dynamic of dissemination of DENV-1 in the Americas has also confirmed the dissemination of this virus from Paraguay to Argentina in 2000 [31]. The DENV-1 of the epidemic of 2000 detected in Paraguay and Argentina grouped in two different lineages [7, 36]. Barrero and colleagues [36] suggested that one lineage of DENV-1 was introduced from Paraguay and the other from Brazil, which is also supported by our phylogenetic analysis (Fig. 5).

The DENV-2 described in this study grouped within the SE Asia/American genotype, which includes most of the DENV-2 isolated in South America and the Caribbean region, in agreement with results obtained in other studies [11, 38–44]. Aquino and colleagues [11] have found the circulation of two DENV-2 lineages in 2005, one including viruses circulated between 2001 and 2005 and the other only viruses from 2005, demonstrating the introduction in Paraguay of a new DENV-2 lineage in that year. The viruses isolated in 2011 in the Asuncion metropolitan area grouped in a different clade where the 2001–2005 viruses were located, indicating the introduction of a new virus lineage in the country in 2011. The monophyletic cluster where the Paraguayan DENV-2 of 2011 were grouped includes viruses isolated in 2008 in Rio de Janeiro, Brazil, as the oldest viruses of this cluster, suggesting this is the origin of the DENV-2 introduced in Paraguay. Besides, this cluster includes viruses isolated in Peru and Bolivia, suggesting the same virus lineage introduced in Paraguay was also disseminated into Peru and Bolivia. A similar phylogenetic relationship of DENV-2 from Paraguay, Peru, Bolivia, and Brazil has been found in a multicenter surveillance program, which includes three DENV-2 isolated in Paraguay in 2010 [45]. Two of the DENV-2 of that study were isolated in Asunción and one in the Department of Alto Parana, located in the East region of the country, in the border with Brazil. The DENV-2 isolated in 2010 was closely related to those isolated in 2011 (Fig. 5), suggesting that only one lineage of this virus was circulating in Paraguay by that time.

## Conclusions

The DENV-1 and DENV-2 were responsible for the dengue epidemic affected Paraguay in 2011. We found strong evidence that the DENV-1 was introduced into Paraguay from Argentina, while the DENV-2 from Brazil, replacing previous virus lineages. Molecular epidemiology studies are of great interest to analyze the dynamic of DENV spread, which are useful for early implementation of containment measures to reduce the risk of explosive epidemics caused by this virus.

## Supplementary Information

**Additional file 1.** Alignment of DENV-1. The E gene sequences of the DENV-1 (n = 15) obtained in this study were aligned with the E gene sequences of representative DENV-1 (n = 381) isolated worldwide. The alignment was performed with the CLC Main Workbench software (QIAGEN, USA).

**Additional file 2.** Alignment of DENV-2. The E gene sequences of the DENV-2 (n = 6) obtained in this study were aligned with the E gene sequences of representative DENV-1 (n = 702) isolated worldwide. The alignment was performed with the CLC Main Workbench software (QIAGEN, USA).

## Data Availability

Protein E gene sequences generated in this study were deposited in the GenBank, under the Accession numbers: KF419412-32. All other data generated or analyzed during this study are included in this published article and its additional information files.

## Abbreviations

DENV: *Dengue virus*
DENV-1 to 4: *Dengue virus* Type 1 to 4
C: Capsid protein
prM: Pre-membrane protein
NS: Non-structural protein
CH-ISW: Central Hospital of the Institute of Social Welfare
RT-PCR: Reverse transcription-polymerase chain reaction
BLAST: Basic Local Alignment Search Tool
HSRI: Health Sciences Research Institute
UNA: National University of Asuncion

## References

[1] Bhatt S, Gething PW, Brady OJ, Messina JP, Farlow AW, Moyes CL, Drake JM, Brownstein JS, Hoen AG, Sankoh O (2013). The global distribution and burden of dengue. Nature.

[2] Hammon WM, Rudnick A, Sather GE (1960). Viruses associated with epidemic hemorrhagic fevers of the Philippines and Thailand. Science.

[3] Sabin A (1950). The dengue group of viruses and its family relationships. Bacteriol Rev.

[4] Sweet B, Sabin A (1954). Properties and antigenic relationships of hemagglutinins associated with the dengue viruses. J Immunol.

[5] WHO (2014). Dengue and severe dengue.

[6] Pereira Y, Samudio M, Ojeda A, Cabello Á (2015). Seroprevalence of dengue infection in a district of the Paraguayan Chaco: Population based study. Rev Chilena Infectol.

[7] Avilés G, Rowe J, Meissner J, Manzur Caffarena J, Enria D, St Jeor S (2002). Phylogenetic relationships of dengue-1 viruses from Argentina and Paraguay. Arch Virol.

[8] Rico-Hesse R (1990). Molecular evolution and distribution of dengue viruses type 1 and 2 in nature. Virology.

[9] Benítez-Leite S, Machi M, Gibert E, Rivarola K (2002). Conocimientos, actitudes y prácticas acerca del dengue en un barrio de Asunción. Rev Chil Pediatr.

[10] PAHO (2001). 2001: Number of reported cases of dengue & dengue hemorrhagic fever (DHF), region of the Americas (by country and subregion).

[11] Aquino JD, Tang WF, Ishii R, Ono T, Eshita Y, Aono H, Makino Y (2008). Molecular epidemiology of dengue virus serotypes 2 and 3 in Paraguay during 2001–2006: the association of viral clade introductions with shifting serotype dominance. Virus Res.

[12] PAHO (2003). 2002: Number of reported cases of dengue & dengue hemorrhagic fever (DHF), region of the Americas (by country and subregion).

[13] Aquino VH, Anatriello E, Gonçalves PF, da Silva EV, Vasconcelos PF, Vieira DS, Batista WC, Bobadilla ML, Vazquez C, Moran M (2006). Molecular epidemiology of dengue type 3 virus in Brazil and Paraguay, 2002–2004. Am J Trop Med Hyg.

[14] Alfonso HL, Amarilla AA, Gonçalves PF, Barros MT, de Almeida FT, Silva TR, da Silva EV, Nunes MT, Vasconcelos PF, Vieira DS (2012). Phylogenetic relationship of dengue virus type 3 isolated in Brazil and Paraguay and global evolutionary divergence dynamics. Virol J.

[15] Amarilla AA, de Almeida FT, Jorge DM, Alfonso HL, de Castro-Jorge LA, Nogueira NA, Figueiredo LT, Aquino VH (2009). Genetic diversity of the E protein of dengue type 3 virus. Virol J.

[16] Araújo J, Nogueira R, Schatzmayr H, Zanotto P, Bello G (2008). Phylogeography and evolutionary history of dengue virus type 3. Infect Genet Evol.

[17] PAHO (2007). 2007: Number of reported cases of dengue & dengue hemorrhagic fever (DHF). Region of the Americas (by country and subregion).

[18] Matheus S, Meynard JB, Lavergne A, Girod R, Moua D, Labeau B, Dussart P, Lacoste V, Deparis X (2008). Dengue-3 outbreak in Paraguay: investigations using capillary blood samples on filter paper. Am J Trop Med Hyg.

[19] PAHO (2012). Number of reported cases of dengue and severe dengue (SD) in the Americas, by Country: figures for 2011 (to week noted by each country).

[20] Díaz MB, Hidalgo E, Gamarra NAUn. PARAGUAY. Proyección de la población por sexo y edad,según distrito, 2000–2025. In: October edn. Fernando de la Mora, Paraguay: Dirección General de Estadística, Encuestas y Censos de la Secretaría Técnica de Planificación de la Presidencia de la República.; 2015.

[21] Dos Santos HW, Poloni TR, Souza KP, Muller VD, Tremeschin F, Nali LC, Fantinatti LR, Amarilla AA, Castro HL, Nunes MR (2008). A simple one-step real-time RT-PCR for diagnosis of dengue virus infection. J Med Virol.

[22] Christenbury J, Aw P, Ong S, Schreiber M, Chow A, Gubler D, Vasudevan S, Ooi E, Hibberd M (2010). A method for full genome sequencing of all four serotypes of the dengue virus. J Virol Methods.

[23] Afgan E, Baker D, Batut B, van den Beek M, Bouvier D, Cech M, Chilton J, Clements D, Coraor N, Grüning BA (2018). The Galaxy platform for accessible, reproducible and collaborative biomedical analyses: 2018 update. Nucleic Acids Res.

[24] Nguyen LT, Schmidt HA, von Haeseler A, Minh BQ (2015). IQ-TREE: a fast and effective stochastic algorithm for estimating maximum-likelihood phylogenies. Mol Biol Evol.

[25] Minh BQ, Nguyen MA, von Haeseler A (2013). Ultrafast approximation for phylogenetic bootstrap. Mol Biol Evol.

[26] Cabello Á (2011). Boletín epidemiológico y semanal.

[27] DGEEC (2015). Paraguay: Población nacional estimada y proyectada, según sexo, departamento, y distrito, 2000–2025.

[28] Goncalvez A, Escalante A, Pujol F, Ludert J, Tovar D, Salas R, Liprandi F (2002). Diversity and evolution of the envelope gene of dengue virus type 1. Virology.

[29] Chen R, Vasilakis N (2011). Dengue–quo tu et quo vadis?. Viruses.

[30] Twiddy S, Farrar J, Vinh Chau N, Wills B, Gould E, Gritsun T, Lloyd G, Holmes E (2002). Phylogenetic relationships and differential selection pressures among genotypes of dengue-2 virus. Virology.

[31] de Bruycker-Nogueira F, Mir D, Dos Santos FB, Bello G (2016). Evolutionary history and spatiotemporal dynamics of DENV-1 genotype V in the Americas. Infect Genet Evol.

[32] Pinhánez G (2008). Boletín epidemiológico semanal.

[33] Allende I (2009). Boletín epidemiológico semanal.

[34] Tittarelli E, Mistchenko AS, Barrero PR (2014). Dengue virus 1 in Buenos Aires from 1999 to 2010: towards local spread. PLoS ONE.

[35] Seijo A (2009). Dengue 2009: chronology of an epidemic. Arch Argent Pediatr.

[36] Barrero P, Mistchenko A (2004). Complete genome sequencing of dengue virus type 1 isolated in Buenos Aires, Argentina. Virus Res.

[37] Barrero P, Mistchenko A (2008). Genetic analysis of dengue virus type 3 isolated in Buenos Aires, Argentina. Virus Res.

[38] Bennett SN, Holmes EC, Chirivella M, Rodriguez DM, Beltran M, Vorndam V, Gubler DJ, McMillan WO (2006). Molecular evolution of dengue 2 virus in Puerto Rico: positive selection in the viral envelope accompanies clade reintroduction. J Gen Virol.

[39] Dettogni RS, Louro ID (2012). Phylogenetic characterization of Dengue virus type 2 in Espírito Santo, Brazil. Mol Biol Rep.

[40] McElroy KL, Santiago GA, Lennon NJ, Birren BW, Henn MR, Muñoz-Jordán JL (2011). Endurance, refuge, and reemergence of dengue virus type 2, Puerto Rico, 1986–2007. Emerg Infect Dis.

[41] Méndez JA, Usme-Ciro JA, Domingo C, Rey GJ, Sánchez JA, Tenorio A, Gallego-Gomez JC (2012). Phylogenetic reconstruction of dengue virus type 2 in Colombia. Virol J.

[42] Oliveira MF, Galvao Araujo JM, Ferreira OC, Ferreira DF, Lima DB, Santos FB, Schatzmayr HG, Tanuri A, Ribeiro Nogueira RM (2010). Two lineages of dengue virus type 2, Brazil. Emerg Infect Dis.

[43] Roca Y, Baronti C, Revollo RJ, Cook S, Loayza R, Ninove L, Fernandez RT, Flores JV, Herve JP, de Lamballerie X (2009). Molecular epidemiological analysis of dengue fever in Bolivia from 1998 to 2008. Vector Borne Zoonotic Dis.

[44] Romano CM, de Matos AM, Araújo ES, Villas-Boas LS, da Silva WC, Oliveira OM, Carvalho KI, de Souza AC, Rodrigues CL, Levi JE (2010). Characterization of Dengue virus type 2: new insights on the 2010 Brazilian epidemic. PLoS ONE.

[45] Cruz CD, Forshey BM, Juarez DS, Guevara C, Leguia M, Kochel TJ, Halsey ES (2013). Molecular epidemiology of American/Asian genotype DENV-2 in Peru. Infect Genet Evol.

